# Persistent nasal methicillin-resistant staphylococcus aureus carriage in hemodialysis outpatients: a predictor of worse outcome

**DOI:** 10.1186/1471-2369-14-93

**Published:** 2013-04-23

**Authors:** Holger Schmid, Andre Romanos, Helmut Schiffl, Stephan R Lederer

**Affiliations:** 1 KFH Nierenzentrum Muenchen – Laim, Elsenheimerstr. 36, Munich, 80687, Germany; 2 Clinic and Policlinic IV, Section of Nephrology, University of Munich, Munich, Germany

**Keywords:** Hemodialysis, ESRD, Staphylococcus aureus, MRSA, Comorbidity, Outcome

## Abstract

**Background:**

Nasal colonization with methicillin-resistant *Staphylococcus aureus* (MRSA) is a well defined risk factor for subsequent bacteremia and death in various groups of patients, but its impact on outcome in patients receiving long-term hemodialysis (HD) is under debate.

**Methods:**

This prospective interventional cohort study (performed 2004 to 2010) enrolled 289 HD outpatients of an urban dialysis-unit. Nasal swab cultures for MRSA were performed in all patients upon first admission, at transfer from another dialysis facility or readmission after hospitalisation. Nasal MRSA carriers were treated in a separate ward and received mupirocin nasal ointment. Concomitant extra-nasal MRSA colonization was treated with 0.2% chlorhexidine mouth rinse (throat) or octenidine dihydrochloride containing antiseptic soaps and 2% chlorhexidine body washes (skin). Clinical data and outcome of carriers and noncarriers were systematically analyzed.

**Results:**

The screening approach identified 34 nasal MRSA carriers (11.7%). Extra-nasal MRSA colonization was observed in 11/34 (32%) nasal MRSA carriers. History of malignancy and an increased Charlson Comorbidity Index were significant predictors for nasal MRSA carriers, whereas traditional risk factors for MRSA colonization or markers of inflammation or malnutrition were not able to discriminate. Kaplan-Meier analysis demonstrated significant survival differences between MRSA carriers and noncarriers. Mupirocin ointment persistently eliminated nasal MRSA colonization in 26/34 (73.5%) patients. Persistent nasal MRSA carriers with failure of this eradication approach had an extremely poor prognosis with an all-cause mortality rate >85%.

**Conclusions:**

Nasal MRSA carriage with failure of mupirocin decolonization was associated with increased mortality despite a lack of overt clinical signs of infection. Further studies are needed to demonstrate whether nasal MRSA colonization represents a novel predictor of worse outcome or just another surrogate marker of the burden of comorbid diseases leading to fatal outcome in HD patients.

## Background

Despite great technological advances in hemodialysis (HD) therapy mortality rates of HD patients remain unsatisfactorily high [1]. Next to cardiovascular diseases, infections are major causes of morbidity, hospitalization and mortality in this population. *Staphylococcus aureus* (*S. aureus*) accounts for the majority of bacterial infections in HD patients and has become increasingly resistant to antibiotics worldwide [2].

Elevated Methicillin-resistant *S. aureus* (MRSA) colonization rates are well recognized in long-term HD patients and are associated with a high risk of blood stream infections (BSI) [3,4]. Particularly vascular catheter access represents a primary risk for BSI in HD patients [5].

Colonization of *S. aureus* in the anterior nares hazards patients for subsequent infection of endogenous origin [6]. HD patients at risk for nasal MRSA carriage are particularly those with higher age (≥75 years), prolonged hospitalization, a history of repeated antibiotic administrations and proximity to others with MRSA colonization [7]. Recently Lai and colleagues demonstrated an association between nasal MRSA carriage and poor clinical outcomes in HD outpatients [8].

Comorbidity is another known risk factor for antibiotic-resistant bacterial infections [9]. Although the presence of comorbid conditions can be graded using standardized indexes, clinicians normally simply record their presence. The Charlson Comorbidity index (CCI) was originally designed as a measure for 1-year mortality attributable to comorbidity in hospitalized patients and has been shown to be a prognostic indicator in the treatment of many types of cancer [10].

Timely recognition and isolation of HD patients colonized with MRSA in combination with a stringent decolonization regimen could be a feasible strategy to minimize MRSA transmission rates [7,11]. MRSA colonization represents an undisputed challenge for dialysis units and few studies so far have addressed the clinical consequences of MRSA nasal carriage in ambulatory HD patients [7].

Confronted with the question “MRSA: total war or tolerance?” we implemented a comprehensive infection control practice based on the concept of *“Search-Destroy-and- Follow”* in our urban outpatient dialysis centre. Our preliminary pilot study revealed the clinical relevance of longitudinal MRSA screening, isolating HD and eradication therapy with mupirocin ointment for effective prevention of MRSA bacteremia [12].

Based on these initial findings a prospective interventional surveillance study was performed over 7 years to (i) confirm the clinical epidemiology and risk profile of MRSA carriers, (ii) to test the effectiveness of a systematic MRSA screening and decolonization approach and, most important, (iii) to characterize the outcome of MRSA carriers requiring chronic HD in an outpatient setting.

## Methods

### Study population and design

This prospective interventional cohort study was performed in an urban non-profit dialysis-unit consisting of two wards with a total of 34 beds including a separated four-bed isolation room. From January 2004 to December 2010 a total of 289 outpatients receiving maintenance HD were enrolled. All participants gave their informed written consent before study entry. The analysis was carried out according to the guidelines of the Declaration of Helsinki Principles and anonymity of included patients was strictly preserved. The study protocol was approved from the Ethical Comitee of the University of Munich (Project Nr 527–12).

At study entry, patient’s clinical baseline characteristics including major comorbid disorders (malignancy, cardiac, pulmonary and peripheral arterial occlusive disease, insulin dependent diabetes mellitus IDDM, chronic HCV infection), current immunosuppressive treatment and type of vascular HD access were collected from medical records by two independent investigators. Malignancy was defined as having a history of any malignancy. Suffering from an active malignant disease was specified as an exclusion criterion. The Charlson Comorbidity index (CCI) as well as the age adjusted CCI were calculated using the ICD-10 database information [10].

Generally, longitudinal laboratory data (total blood count, total serum protein, CRP, ferritin, iPTH and HbA1c) were collected routinely in 6-weeks intervals or shorter. For the present study, the most recent results at the date the swabs were taken, were recorded and analysed. Follow-up time was defined as the timespan between date of first swab (study entry) and the time-point when the patient definitely left the center (because of death or transfer to another dialysis facility) or the study ended (December 2010).

In brief, initial routine screening for nasal MRSA carriage was performed in all current patients at the time of study initiation and in all patients that were newly admitted during the study period. Routine screening for nasal MRSA carriage included so called “holiday dialysis patients” (defined as patients from external dialysis facilities that were treated in our unit during their stay in Munich). Follow-up screening nasal swabs during the study period were taken from (i) readmitted patients after hospitalisation, (ii) patients who returned from holiday, and (iii) patients who had assumed contact to MRSA positive patients in the dialysis unit.

Those patients who had at least one positive nasal MRSA culture were defined as MRSA carriers, whereas all patients with persistent negative MRSA culture results were classified as noncarriers. The primary end point of study was death from any cause.

### Microbiology

Swabs were taken from the anterior nares of the nose by the attending nephrologists as described [12]. Additional swabs of throat and skin areas (forehead, ears, axillae, hands) were taken from patients with documented nasal MRSA carriage.

After transfer to the laboratory, swabs were placed on mannitol salt agar and incubated at 37°C for 48 hours. Mannitol fermenting colonies were selected and subcultured to trypticase soy agar and 5% sheep blood agar plates and incubated at 37°C. Identification of *S. aureus* was based on colony morphology, DNase production and latex agglutination as described. *S. aureus* isolates were screened for methicillin resistance following the National Committee for Clinical Laboratory standards (NCCLS) disk-diffusion method. Overnight cultures from sheep blood agar plates were plated on Mueller-Hinton agar and a 1 μg oxacillin disk was placed on the inoculated plate. Zone diameters were measured and recorded after 24 hour incubation at 37°C as sensitive (greater than 13 mm) or resistant (less than 10 mm).

### Decolonization therapy for nasal MRSA carriers and extra-nasal MRSA colonization

Patients with nasal MRSA colonization were treated in a separate ward (isolating HD) and received topical mupirocin ointment thrice daily for five days. Additional MRSA carriage at other sites was accessorily treated as follows. Patients with MRSA throat colonization received 0.2% chlorhexidine mouth rinse thrice daily for five days. Patients with positive MRSA skin swabs (forehead, ears, axillae, hands) were treated with antiseptic octenidine dihydrochloride containing soaps and 2% chlorhexidine body washes once daily for five days [13,14]. An explanatory note detailing the specific decolonization techniques together with the prescription for the relevant antiseptic agent(s) (mupirocin ointment, 0.2% chlorhexidine mouth rinse, 2% chlorhexidine body wash and antiseptic octenidine dihydrochloride containing soap, respectively) was therefore directly delivered to the patient, to family members of carriers or, in the case of nursing home residents, to the nursing staff respectively.

Follow-up nasal swabs were taken from nasal MRSA carriers three to five days after completing nasal mupirocin ointment. If this swab was negative for MRSA, subsequent nasal swabs on the following two dialysis sessions were performed. A series of three negative results was defined as successful nasal MRSA decolonization. For confirmation of MRSA eradication additional nasal swabs were taken ten days after the third negative swab, as well as one, three and twelve months after the end of decolonization procedures. Persistent nasal carriage was defined by two or more consecutive positive nasal swab cultures for MRSA.

### Statistical analysis

Characteristics between the groups (MRSA carriers or noncarriers) were compared using the Kruskal-wallis test or the Mann–Whitney U- test, as indicated. A logistic regression model was used for analyzing associated clinical factors of nasal MRSA carriage. Kaplan-Meier estimates were used to obtain the proportion of patients who died of any cause during follow-up. Longitudinal laboratory parameters were assessed with ANOVA. All tests were two-tailed with significance defined by p values <0.05. Statistical Package for the Social Sciences (SPSS) for Windows Version 8.0 (SPSS Inc; Chicago, IL, USA) and MedCalc 11.0.4 software was used for statistical analysis.

## Results

### Clinical epidemiology of nasal MRSA colonization

**Figure 1:**
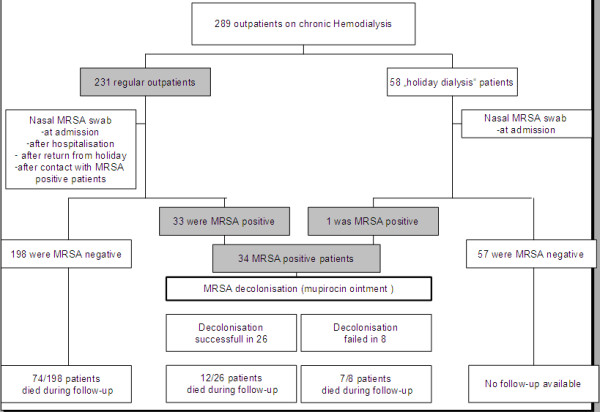
**MRSA screening and outcome results in 289 HD outpatients.** All prevalent outpatients that gave informed written consent and did not suffer from an active malignant disease (exclusion criterion) were enrolled. The 289 patients represent approximately 98% of all eligible outpatients that were treated during the study period in our dialysis unit.

289 chronic dialysis patients, representing approximately 98% of all eligible outpatients that were treated during the study period, were enrolled. A total of 1562 nasal swab cultures were obtained. Detailed screening and outcome results are summarized in Figure 1. The average, median, standard deviation (SD), and range number of nasal MRSA cultures obtained per patient for routine screening were 5.03, 4.00, 4.50, and 1–23, respectively. 204 of the 1562 nasal swabs were performed in nasal MRSA carriers to control efficacy of decolonization.

The screening approach identified 34 nasal MRSA carriers (11.7%). Prevalence of nasal MRSA carriage was 8.3% for men and 3.4% for women. The majority of MRSA positive patients (24/34, 70.5%) were supported by nursing services or even were nursing home residents. Median duration of follow-up was comparable between nasal MRSA carriers and noncarriers (mean follow up ± SD in months, 36 ± 23 vs. 29.5 ± 21, *p* = 0.11).

Concomitant extra-nasal MRSA colonization of throat and/or skin was observed in 11 (32%) of these 34 nasal MRSA carriers. Throat swabs were positive in four patients, swabs from skin sites were positive in five patients, and two patients tested positive in swabs from skin and throat.

### Clinical risk factors for nasal MRSA colonization and comparison of laboratory data

Clinical characteristics that have been identified in previous studies as risk factors for MRSA colonization in HD patients (age, IDDM, type of vascular access, cardiac and pulmonary comorbidities, presence of peripheral arterial occlusive disease) did not significantly differ between nasal MRSA carriers and noncarriers (Table 1).

**Table 1:** Clinical features and laboratory parameters of 289 HD patients

**Clinical characteristics**	**Noncarriers MRSA negative (n = 255)**	**Carriers MRSA positive (n = 34)**	**Level of significance*p***
Age (mean; median years)	62.0; 65.0	67.3; 70.0	0.055
Insulin dependent Diabetes melitus (n,%)	95, 37.3	12, 35.3	0.446
HD access permanent catheter (n,%)	44, 17.2	5, 14.7	0.649
Coronary heart disease (n,%)	206, 80.8	29, 85.3	0.265
Peripheral art. occlusive disease (n,%)	57, 22.4	12, 35.3	0.272
Pulmonary disease (n,%)	46, 18.0	2, 5.9	0.131
Leukocytes (k/μl)	7.70 ± 2.55	8.58 ± 2.57	0.075
CRP (mg/dl)	1.79 ± 3.15	1.74 ± 2.44	0.935
Ferritin (ng/ml)	500 ± 588	676 ± 536	0.124
Total Serum Protein (g/dl)	6.66 ± 0.57	6.70 ± 0.67	0.751

Representative clinical risk factors and comorbidities as well as typical laboratory markers for inflammation or malnutrition showed no significant differences between MRSA carriers and noncarriers.

A total of 64 patients had a history of malignancy, 12 out of 34 nasal MRSA carriers (35.3%) and 52 out of 255 (20.4%) noncarriers, respectively. This difference was statistically significant. Chronic active HCV infection (2/34 vs. 4/255, *p* = 0.134) or current immunosuppressive treatment (3/34 vs. 9/255, *p* = 0.165) did not significantly differ between both groups.

Comparison of representative laboratory markers for inflammation (e.g. leukocytes, CRP, ferritin) or malnutrition (e.g. total serum protein), recorded at the date when the swab cultures were obtained, revealed no significant differences between MRSA carriers and noncarriers (Table 1).

### Age adjusted Charlson Comorbidity index and outcome analysis

**Figure 2:**
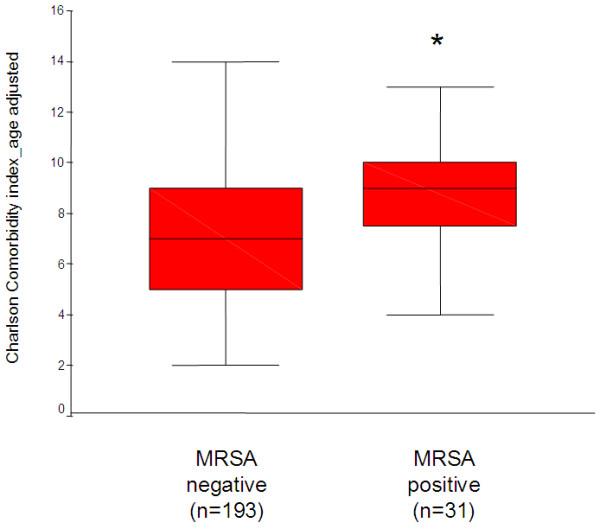
**Charlson Comorbidity index in nasal MRSA carriers and noncarriers.** The age adjusted Charlson Comorbidity index showed significant differences between 31 nasal MRSA carriers and 193 noncarriers, where sufficient clinical data were available for analysis. Box-and-whisker plots indicating the median of the data set (middle line), the lower and upper quartiles (bottom and top of the box), and the range of values (whiskers) are showed.

In contrast to individual comorbidities (see Table 1) application of the age adjusted Charlson Comorbidity index (CCI) demonstrated significant differences between nasal MRSA carriers and noncarriers (median CCI [interquartile range], 7 [2-14] vs. 9 [4-13], p < 0.001) (Figure 2). The CCI as well as the age adjusted CCI were independent of the patients gender.

This analysis was performed in 31/34 MRSA positive patients and in 193/198 MRSA negative regular outpatients, where detailed clinical charts were available for review. Due to the lack of sufficient clinical information and to avoid a systematic selection bias 57/58 “holiday dialysis” patients that were not permanently treated in the dialysis facility had to be excluded.

**Figure 3:**
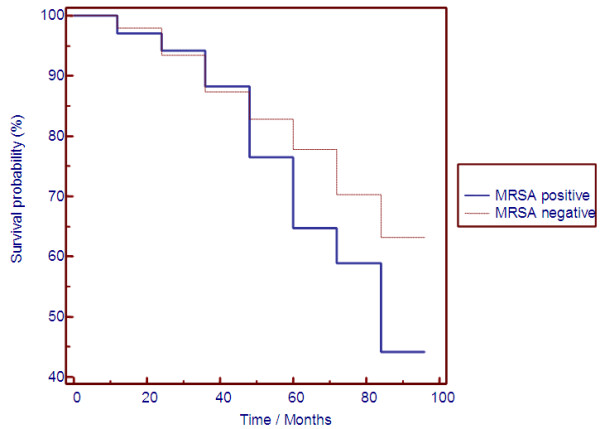
**Kaplan-Meier analysis in nasal MRSA carriers and noncarriers.** Kaplan-Meier analysis showed significant survival differences between 34 nasal MRSA carriers and 198 noncarriers for all cause mortality. Of note, differences between both groups do not appear until after several years of follow-up.

During follow-up 74/198 (37.4%) MRSA negative outpatients and 19/34 (55.9%) MRSA positive patients died. Causes of death are summarized in Table 2. Sepsis was declared as cause of death in 26% of MRSA negative and 21% of MRSA positive patients, respectively. Kaplan-Meier analysis showed survival differences between nasal MRSA carriers and noncarriers with a significantly increased probability for all cause mortality in nasal MRSA carriers (Figure 3).

**Table 2:** Causes of death in MRSA carriers and noncarriers

**Cause of death**	**Noncarriers MRSA negative (n = 74)**	**Carriers MRSA positive (n = 19)**	**Level of significance*p***
**Cardiovascular disease (n;%)**	20; 27	4; 21	0.154
**Respiratory disease (n;%)**	4; 9	5; 27	0.354
**Sepsis (n;%)**	19; 26	4; 21	0.162
**Cerebrovascular disease (n;%)**	6; 8	0; 0	0.141
**Cancer (n;%)**	5; 7	1; 5	0.180
**Bleeding (n;%)**	3; 4	1; 5	0.197
**Not specified (n;%)**	14; 19	4; 21	0.205

Absolute patient numbers (n) and percentages are depicted. Differences between both groups with regard to causes of death were not significant.

### Detailed analysis of decolonization success and outcome in nasal MRSA carriers

For a further detailed analysis we compared the 34 MRSA positive patients with regard to (i) effective or failed persistent nasal MRSA decolonization and (ii) outcome (Table 3).

**Table 3:** Comparison of 34 MRSA positive patients with regard to (i) effective or failed decolonization and (ii) outcome

**Longitudinal differences**	**Successful decolonization (n = 26)**	**Decolonization failed (n = 8)**	**Level of significance*P***	**Survivors (n = 15)**	**Non-survivors (n = 19)**	**Level of significance*P***
CRP (mg/dl)	- 0.53	5.77	0.004	- 0.15	1.42	n.s.
Hb (g/dl)	0.53	2.30	n.s.	0.67	−0.57	n.s.
Leukocytes (k/μl)	- 0.39	2.43	n.s.	0.43	0.16	n.s.
**Basal** CCI age adjusted	8.40	9.80	n.s.	7.10	9.80	0.006
**Additional** Extra-nasal MRSA (n;%)	8; 31	3; 38	n.s.	5; 33	6; 31	n.s.

Longitudinal laboratory differences (CRP, Hb and leukocyte count), the age adjusted CCI and results of additional extra-nasal swabs (throat and skin) for MRSA at baseline are summarized.

**Figure 4:**
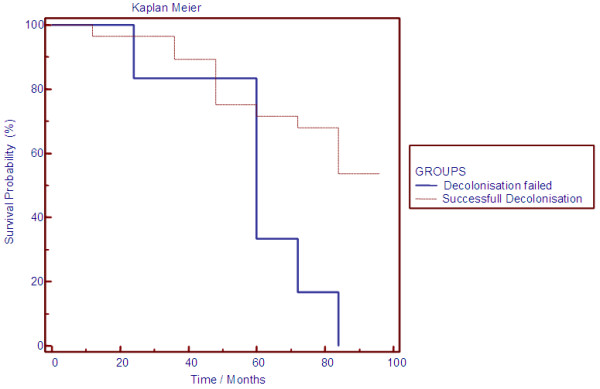
**Survival differences between patients with successful MRSA decolonization and persistent nasal MRSA carriers.** Kaplan-Meier analysis showed significant survival differences between patients with successful MRSA decolonization and persistent MRSA carriers with failure of this approach. Of note, differences between both groups do not appear until after several years of follow-up.

(i) Repeated follow-up swabs demonstrated that our decolonization strategy with mupirocin ointment was effective for persistent eradication of nasal MRSA colonization in 26/34 (76.5%) patients, but failed in 8 patients. A significant longitudinal increase in CRP levels was obvious for patients with failure of the decolonization strategy. The age adjusted CCI did not significantly differ between both groups.

(ii) During follow-up 12/26 (46.1%) patients died after successful MRSA decolonization. MRSA carriers with failure of the MRSA eradication approach had an extremely poor prognosis with an increased all-cause mortality (87.5%). Kaplan-Meier analysis for both groups is summarized in Figure 4. A higher age adjusted CCI (median CCI [interquartile range], 9.8 [8-12] vs. 7.1 [3-12], p < 0.006) was significantly associated with death in MRSA carriers. MRSA positive patients who died during follow-up, showed a longitudinal moderate but not significant increase of inflammation markers (CRP and leukocytes) and a decline in Hb values.

Additional extra-nasal MRSA colonization (throat, skin) had no significant impact on nasal decolonization success or survival of nasal MRSA carriers.

## Discussion

Methicillin-resistant *Staphylococcus aureus* (MRSA) remains a pathogen of crucial public health importance, although MRSA colonization and bacteremia rates in patients requiring long term dialysis have been observed to be decreasing in the United Kingdom [15]. Our prospective investigation identified 34 (11.7%) nasal MRSA carriers in a cohort of 289 HD outpatients of an urban dialysis-unit. This prevalence rate is in accordance with results of a prior pilot study [12] and in line with data from other European surveys [16,17], even though prevalence varies due to differences in populations and geographic areas studied, as well as due to different sensitivity of diagnostic procedures [18].

Higher age and prior hospitalization are the most acknowledged risk factors for MRSA colonization in patients requiring chronic HD [17,19]. Frequently noted other risk factors are a permanent tunneled cuffed dialysis catheter, chronic obstructive lung disease, congestive heart failure, or a sepsis history [17-21]. In addition, protein malnutrition (i.e. low serum albumin) may increase the patients` susceptibility to MRSA colonization [19].

In our cohort neither often-quoted single risk factors for nasal MRSA colonization such as age, type of vascular access, comorbid disorders other than a history of malignancy, nor markers of inflammation or malnutrition had the statistical power to discriminate between MRSA carriers and noncarriers. However, an increased age adjusted CCI was an independent predictor for nasal MRSA colonization. These findings support the hypothesis that not individual comorbidities or laboratory markers but rather an aggregate measure of a person’s risk due to comorbid conditions characterizing an impaired general state of health could serve as a significant predictor for MRSA colonization in HD outpatients.

Early systematic screening of all patients entering the dialysis unit combined with the option of decolonizing carriers is considered to be an essential pillar of any MRSA control programme [22]. Eradication of MRSA carriage has generally been difficult, and currently the routine use of decolonization for non-surgical patients including HD patients with nasal MRSA colonization, is not supported by data [23,24]. Mupirocin has been used as a topical antibiotic applied to the nares and tunnel exit sites of venous catheters HD patients to reduce *S. aureus* colonization, but long-term success of this strategy is unclear. Here mupirocin ointment was able to eradicate nasal MRSA colonization in approximately 75% of carriers, documenting a significant benefit of this strategy in an outpatient dialysis centre. Concomitant extra-nasal MRSA colonization in nasal MRSA carriers had no influence on nasal decolonization failure or outcome. Due to the small sample size and the low prevalence of extra-nasal MRSA colonization, the impact of the applied extra-nasal decolonization approaches (0.2% chlorhexidine mouth rinse for throat decolonization or octenidine dihydrochloride containing antiseptic soaps and 2% chlorhexidine body washes for skin decolonization) remains unclear. Of note, the important value of extra-nasal testing for MRSA, particularly when combined with decolonization, was unknown at the time of study initiation [25].

In general, subjects with a high burden of comorbid disorders are more susceptible to antibiotic-resistant bacteria than the general population, although the distribution and magnitude of comorbid diseases known as risk factors for acquiring MRSA and Methicillin-sensitive *S. aureus* (MSSA) were comparable in a pooled analysis of community- and hospital-onset cases [26]. Various reports described a robust correlation of CCI scores and MRSA infection with consecutive bacteremia in different cohorts [27,28]. In contrast, data evaluating the association between CCI score and nasal MRSA carriage are scarce, and only a single report in long-term-care facility residents identified a high CCI as a significant risk factor for nasal colonization [29].

The incidence of invasive MRSA infections is estimated 100 times higher among dialysis patients than in the general population (45 vs. 0.4 per 1000 patients) [30], and a robust association of MRSA BSI with worse outcome is established in hospitalized patients. Also MRSA colonization is associated with subsequent MRSA infection and an increased all-cause mortality among e.g. ICU patients [31], comparable data for HD outpatients are lacking.

A recent report demonstrated the association between MRSA colonization and poor clinical outcomes in HD outpatients, with a 2.5 fold increased risk for all-cause mortality in nasal MRSA carriers compared to noncarriers [8]. Here we were able to establish a direct link between elevated age adjusted CCI, nasal MRSA colonization and increased all-cause mortality in HD outpatients, indicating that nasal MRSA carriage is not an immanent predictor of death by bacterial sepsis in this population but rather serves as a surrogate marker for a poor general condition. This finding suggests that a distinct combination of comorbidities could result in a positive MRSA carriage status and a worse outcome in nasal carriers, but additional studies are needed to confirm these results.

There are some limitations to our study, notably concerning microbiological technique, decolonization approach, and study design.

First, identification of MRSA and determination of antibiotic susceptibilities was performed according to local protocols as recently described [12]. We did not use commercial PCR-based methods or molecular assays to identify MRSA carriers, even though several studies have shown a higher sensitivity for these tests [32]. Albeit an increased proportion of infections with CA-MRSA strains is documented in the dialysis population [33], we have not differentiated MRSA isolates into community-acquired (CA-MRSA) and healthcare-associated (HA-MRSA) strains, as currently the impact of CA-MRSA strains on mortality in HD patients is unclear [34] and former genetic analysis of the isolated strains did not reveal CA-MRSA strains [12]. Furthermore, as our study approach did neither include periodical testing for nasal MRSA carriers nor MRSA strain typing, a definite classification of MRSA acquisition was not feasible [35].

Second, we were not able to further analyze the underlying reasons for failure of the decolonization approach with mupirocin in a subset of patients, even though a significant longitudinal increase in CRP levels, probably indicating systemic inflammation, was obvious in this cohort. High-level mupirocin resistance, mediated by a plasmid-encoded mupA gene, has been associated with decolonization failure, and increased resistance rates have been associated with increased mupirocin use [36]. In addition, although decolonization was performed in more than 70% of patients under surveillance of nursing services, we cannot exclude that patients’ nonadherence has limited the success of our approach [37].

Third, the CCI used in our risk factor analysis represents a comorbidity index that was originally designed as a longitudinal measure for mortality in hospitalized patients, but currently no more convenient index has been developed specifically for outcome studies in the outpatient HD population colonized with antibiotic-resistant bacteria [38].

Finally, we used all cause mortality as primary study endpoint, but were not able to clearly determine the rate of MRSA BSI and infection-related deaths in our cohort, as the results of blood cultures drawn after hospital admission were not available for a systematic analysis. However, analyzing the causes of deaths in MRSA carriers and noncarriers, we found even slightly higher rates of sepsis in MRSA negative patients, arguing against the hypothesis that MRSA BSI represent the dominant lethal cause in nasal MRSA carriers. In addition, particularly the first 30 days after an infection-related hospitalization seem to represent a high-risk period for cardiovascular events leading to a potential bias in cause of death analysis [39].

## Conclusion

In conclusion, this study demonstrates that beside well-known predictors of outcome in HD patients, such as cardiovascular diseases or bacterial infections, nasal MRSA carriage with failure of mupirocin decolonization is associated with significant mortality. Based on our findings, however, it remains unclear, whether nasal MRSA colonization could serve “per se” as a novel predictor of outcome or if nasal MRSA colonization is only another surrogate marker for increased comorbidity, as a distinct combination of comorbidities mapped in an age-adjusted CCI results both in a positive MRSA carriage status and a worse outcome. Further studies have now to evaluate, whether a revised comorbidity measure specific for resistant bacteria would likely provide a better assessment of the comorbidity-attributable risk of mortality in HD patients with nasal MRSA colonization.

## Competing interests

The authors declare that they have no competing interest.

## Authors’ contributions

HS, AR, HS and SRL were involved in the design of the study. HS, AR and HS performed the analysis and interpretation of data for this study. HS, HS and SRL were involved in writing the manuscript and revising it critically for important intellectual content. All authors read and approved the final manuscript.

## Pre-publication history

The pre-publication history for this paper can be accessed here:

http://www.biomedcentral.com/1471-2369/14/93/prepub
